# An overview of childhood cancer care and outcomes in Egypt: a narrative review

**DOI:** 10.3332/ecancer.2024.1676

**Published:** 2024-02-28

**Authors:** Ranin Soliman, Nancy Bolous, Carl Heneghan, Jason Oke, Anne-Marie Boylan, Wael Eweida, Sherif Abouelnaga, Alaa Elhaddad

**Affiliations:** 1 Health Economics and Value Unit, Children’s Cancer Hospital 57357 Egypt (CCHE), Cairo 4260102, Egypt; 2 Centre for Evidence-Based Medicine, University of Oxford, OX1 2JD Oxford, UK; 3 School of Life and Medical Sciences, University of Hertfordshire hosted by Global Academic Foundation, New Administrative Capital, Cairo 4813001, Egypt; 4 Department of Global Pediatric Medicine, St Jude Children’s Research Hospital, Memphis, TN 38105, USA; 5 Centre for Evidence-Based Medicine, Nuffield Department of Primary Care Health Sciences, University of Oxford, OX1 2JD Oxford, UK; 6 Chief Operating Office, Children’s Cancer Hospital 57357 Egypt (CCHE), Cairo 4260102, Egypt; 7 Chief Executive Office, Children’s Cancer Hospital 57357 Egypt (CCHE), Cairo 4260102, Egypt; 8 Paediatric Oncology Department, National Cancer Institute, Cairo University, Cairo 11796, Egypt; 9 Paediatric Oncology Department, Children’s Cancer Hospital 57357 Egypt (CCHE), Cairo 4260102, Egypt; † Co-first authors

**Keywords:** childhood cancer, disease burden, health outcomes, treatment costs and cost-effectiveness, global health, evidence synthesis

## Abstract

Childhood cancer is an urgent priority in Egypt, owing to a large number of children with cancer, the great need and demand for paediatric oncology services, limited resources/funds and inferior survival outcomes. Therefore, an overview of the status of childhood cancer care in Egypt and an evidence-based approach towards optimal utilisation of resources/funds to improve this care are needed. This paper summarises key evidence about childhood cancer care and outcomes in Egypt. We conducted a narrative literature review using a structured search strategy of the MEDLINE database through the PubMed interface. All relevant evidence was summarised under five main sub-topics: (1) burden of childhood cancer in Egypt; (2) treatment approaches; (3) health outcomes; (4) costs and cost-effectiveness of treatment; and (5) barriers and facilitators to optimal childhood cancer care. We found high estimates of disease burden of childhood cancer in Egypt. Furthermore, childhood cancer treatment in Egypt is based on either implementing intensity-regulated protocols or adopting international protocols with or without adaptations to local contexts, leading to varying standards of care among the different treating centres. Limited data about the survival outcomes, costs and cost-effectiveness of treatment exist, although high-quality data from retrospective cohort studies were published from a large paediatric oncology centre (Children’s Cancer Hospital Egypt–57357). As Egypt joins the WHO Global Initiative for Childhood Cancers as a focus country, it is prepared to move towards streamlining national efforts to implement a national childhood cancer plan to advance care, improve health outcomes and optimise resource use. Through these efforts, Egypt could become a beacon of hope and a role model to other low- and middle-income countries seeking to improve their childhood cancer care.

## Introduction

Projected estimates anticipate that cancer will develop in 13.7 million children worldwide between 2020 and 2050. Of these patients, 6.1 million will never receive a diagnosis, and 11.1 million will die, with 84% of these deaths occurring in low-income and lower-middle-income countries [1]. Although recent advances in treatments have significantly improved the childhood cancer survival rate in high-income countries to 80%, in low- and middle-income countries (LMICs), it lags at approximately 20% [2]. This stark contrast incentivised the World Health Organisation (WHO) and St. Jude Children’s Research Hospital to launch the Global Initiative for Childhood Cancer (GICC) in 2018, with the goal of increasing the net survival of childhood cancer patients from 20% in 2015 to 60% in 2030 [3].

According to the World Bank classification, Egypt is a lower-middle income country [4], geographically located in Africa, and lies within the WHO Eastern Mediterranean Region (EMR) [5]. Both Africa and the EMR witnessed the highest per capita number of annual childhood cancer cases, with Africa ranking first and the EMR ranking second [6]. Egypt ranks the tenth country globally with the largest number of incident childhood cancer cases, and the second in the EMR, contributing significantly to the global burden [5, 7]. Analysis of the current situation is necessary to effectively design a national cancer control plan (NCCP) to map out an evidence-based approach towards improving childhood cancer treatment outcomes in Egypt. Nonetheless, there is a paucity of data regarding childhood cancer burden, services and outcomes in Egypt.

This paper aims to identify and summarise key evidence about tackling childhood cancer in Egypt, to reflect an overview of the disease burden, health outcomes, treatment approaches and resources used in childhood cancer care, their costs and cost-effectiveness estimates, as well as other factors affecting paediatric oncology care in Egypt.

## Methods

A narrative literature review [8] was conducted in which we performed a structured search of the MEDLINE database through the PubMed interface. Searches covered three topics: 1) childhood cancer disease burden and outcomes (survival) in Egypt; 2) childhood cancer treatment resources, costs and cost-effectiveness in Egypt; and 3) Barriers and facilitators to paediatric oncology care in Egypt (see Table 1 for search terms). Moreover, forward and backward citation to identify all the pertinent literature and grey literature of key organisations, including the WHO and the International Agency for Research on Cancer (IARC), were performed.

All evidence was summarised under five main sub-topics; (1) burden of childhood cancer in Egypt; (2) treatment approaches; (3) health outcomes; (4) costs and cost-effectiveness of treatment; and (5) barriers and facilitators to optimal childhood cancer care.

## Results

### Burden of childhood cancer in Egypt

**Figure 1.**
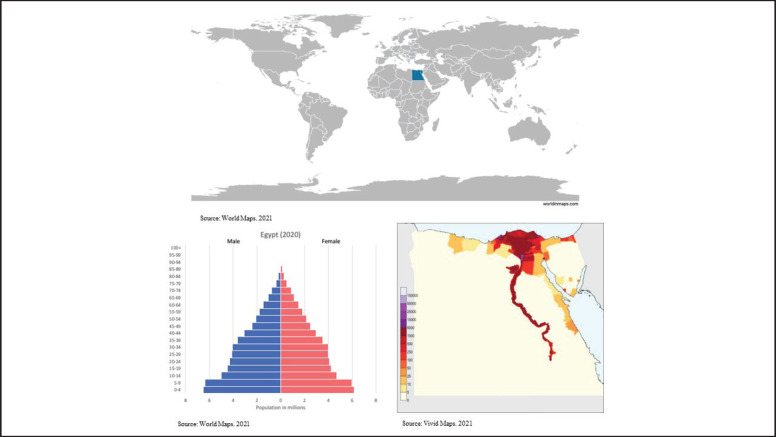
Egypt’s geographical map, age structure and population density.

The WHO estimated in 2020 that the annual number of new childhood cancer cases in Egypt (patients aged 0–14 years) was 6,803 representing 20% of the total number of cases in the region [9–11]. This is attributed to the relatively large population of Egypt (102.3 million in 2020) [12], with 34% of the population under the age of 15 [13]. Figure 1 shows Egypt’s geographical location and age structure.

Based on GLOBOCAN 2020 estimates by the IARC, the childhood cancer incidence rate was 12.1 per 100,000 children at risk in Egypt, which totals 4,181 patients [5, 7]. Moreover, GLOBOCAN estimated that Egypt had the greatest number of annual childhood cancer deaths (*n* = 1,581) in the EMR, with a mortality rate of 4.6 per 100,000 children at risk [5, 7]. Childhood leukaemia accounted for 21.6% of total childhood cancer deaths in Egypt, followed by brain/Central Nervous System tumours (20.5%) and non-Hodgkin lymphoma (12.4%) [5, 7]. Another modelling study by Ward *et al* [14], developed as part of the *Lancet Oncology* Commission on Childhood Cancer, estimated that for the age group 0–14 years, the respective total national incidence and number of diagnosed patients were 7,218 and 4,012 in 2020, and were projected to reach 7,404 and 4,127 in 2030 [14].

Besides using modelled estimates and projections, it is difficult to accurately estimate the burden of childhood cancer in Egypt due to the limited capacity of the cancer registration system [15, 16]. To date, only one study – conducted by Ibrahim *et al* [17] – reported childhood cancer incidence rates in Egypt between 2008 and 2011 using data from the population-based national cancer registry program (NCRP). The researchers estimated an age-specific incidence rate of 17.1, 15.4, 10.5 and 10.5 per 100,000 children aged 0–4, 5–9, 10–14 and 15–19 years, respectively [17].

This study was limited by some deficiencies that are common in cancer burden documentations in LMICs, making it unreliable to estimate cancer incidence rates on regional and national levels. To address these limitations, the authors estimated regional incidence rates by classifying Egypt into three geographical regions (lower, middle and upper), and selecting one governorate to represent each region [17]. Childhood cancer incidence rates were calculated on the regional level using the population-based data available at the NCRP in these three governorates [17]. Afterwards, to determine the national childhood cancer incidence rates, the authors developed a simulation model that used population-based incidence data of the three regions to estimate national incidence rates for the entire country, adjusting for total population demographics [17]. Another limitation of this study is that these estimates are now considered outdated, and no recent data based on population-based cancer registries has been published since then. Nevertheless, despite the limitations of this study, it provided the only available childhood cancer incidence rates based on population-based estimates at the national and regional levels of Egypt [17].

### Treatment approaches in Egypt

In Egypt, there are approximately 20 paediatric oncology units/centres for the treatment of children with cancer, with limited to adequate diagnostic services and adequate availability of medications and radiotherapy [18]. Treatment is either based on implementing intensity-regulated protocols (starting with low-intensity and gradually intensifying based on patients’ tolerability) or following international protocols with or without adaptations to local contexts [16, 19, 20].

**Figure 2.**
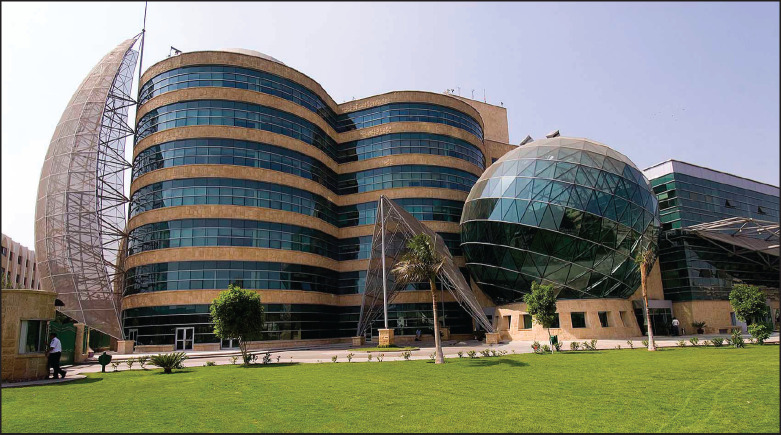
Children’s Cancer Hospital Egypt – 57357 (CCHE).

The Children’s Cancer Hospital Egypt (CCHE) – known as *57357* hospital (shown in Figure 2) – is a sizeable paediatric oncology centre in Egypt with 320 inpatient beds and treats around 2,500–3,000 children (aged 0–18 years old) newly diagnosed cancer annually, representing approximately 40% of all children with diagnosed with cancer across Egypt [16, 21]. CCHE is a non-profit charity hospital that uses philanthropic donations to treat children with cancer for free [21]. Childhood cancer treatment at CCHE follows international protocols such as those of the Children’s Oncology Group, International Society of Paediatric Oncology (SIOP) and St. Jude Total XV for acute lymphoblastic leukaemia (ALL) [19–21]. The hospital provides comprehensive childhood cancer care with state-of-the-art diagnostic and therapeutic services, offering multimodal treatment that includes chemotherapy regimens, specialised surgeries (neurosurgery, orthopaedic surgery, surgical oncology), bone marrow transplant, external beam radiation therapy and cyber knife radiosurgery and is currently installing proton beam therapy [21].

### Childhood cancer health outcomes in Egypt

#### Survival rates in Egypt

Childhood cancer survival cannot be evaluated on a national level in Egypt due to the absence of survival data in the population-based cancer registry [5, 16]. A baseline assessment of paediatric oncology care in Egypt estimated a 5-year survival for children with cancer of around 40%–60% based on modelled data [18].

A previous literature review article by Basbous *et al* [16] summarised the existing literature and abstracts about childhood cancer survival in Egypt, showing that survival ranged from 45% to 70% depending on cancer type, availability of resources, quality of care and adopted treatment protocols, which varied across centres/units in Egypt. Notably, only a few cancer types were included in the review, with no subtype, risk or stage stratification, and or survival trends reported. The findings of this review article of nine published studies and 17 SIOP abstracts show that the 5-year overall survival rate ranged from 50% to >80% for ALL and 50%–65% for solid tumours. The highest survival estimates (>90%) were achieved for Hodgkin lymphoma and retinoblastoma. However, the included studies in the review estimated survival for only a few cancer types using a small sample size, limiting the confidence in the quality and generalisability of evidence [16].

Soliman *et al* [22] conducted a retrospective cohort study using hospital-based registry data from CCHE to determine the survival of all childhood cancers combined and stratified by cancer type/subtype according to the International Classification of Childhood Cancer, 3rd edition (ICCC-3 groups), risk and stage at diagnosis. A cohort of 15,779 patients aged 0–18 years and treated in CCHE between 2007 and 2017 were included in the study. Findings showed that the 5-year overall survival rate was 72.1% for all cancers combined, and the survival trend increased significantly from 69.6% in 2007–2012 to 74.2% in 2013–2017.

Since CCHE is regarded as a centre of excellence for paediatric cancer care in Egypt, it strives to support other paediatric oncology centres in Egypt and the community through several activities and initiatives aimed at improving childhood cancer survival outcomes across Egypt [21]. Such efforts include: (1) capacity building, through running training programs, fellowships and internships at CCHE to train paediatric oncologist, clinical pharmacists, nurses and researchers on the best practices and advances in childhood cancer care and research; (2) unified standard treatment protocols, through sharing the evidence-based standard treatment protocols adopted at CCHE with other paediatric oncology centres in Egypt such as the National Cancer Institute and Nasser Institute for Research and Treatment; (3) diagnostics best practice, through sharing the regimens and roadmaps of best practice for childhood cancer diagnosis with other diagnostic centres; and (4) health promotion, through making awareness campaigns about early detection of cancer in children for the greater good of the community [21].

#### Disease relapse and treatment-related mortality (TRM)

Disease relapse remains a significant challenge to improving childhood cancer survival outcomes, as survival after relapse remains generally poor [23]. Relapse is the most common cause of treatment failure in paediatric ALL and is experienced by around 15%–20% of paediatric patients with ALL; thus, relapsed ALL is considered the fourth most common childhood cancer [23, 24]. Additionally, around 2% of paediatric patients with ALL have refractory disease [24, 25], making relapsed/refractory (r/r) ALL a leading cause of death by cancer in children [26]. Whereas around 30% of children with acute myeloid leukaemia (AML) experience relapse [27]. The survival outcomes of children with r/r ALL or AML remain poor (approximately 40%) [24], requiring intensive treatment using novel drugs.

TRM is becoming an important area for improving overall survival, especially in LMICs where more children with cancer die from treatment-related complications, and fewer die from the disease [28]. TRMs are defined as ‘death occurring in the absence of progressive cancer’ [28]. TRMs are more common in haematological malignancies and are much higher in LMICs with suboptimal supportive care [28, 29]. A systematic literature review representing 68,351 childhood cancer patients treated in LMICs concluded that TRM accounted for 30.9% of overall mortality, with trends improving with time in upper-middle income countries but remaining unchanged in low-income and lower-middle-income countries [30].

In Egypt, early deaths and TRMs are a vital concern during intensive induction therapy in paediatric ALL and AML, where 47.3% of all deaths occur [31]. The majority of the early deaths (65%) are caused by infection [32].

### Costing and cost-effectiveness in Egypt

#### Resource use and costs

Given the large number of children with cancer in Egypt, there is a high need and demand for paediatric oncology services despite the limited resources and funds. It is, therefore, essential to maximise the use of the available resources and manage costs effectively to accommodate the need for services. A retrospective cohort study was conducted by Soliman *et al* [33] to assess the patterns and trends in resource use and costs of childhood cancer treatment and the associated factors, using data from 8,886 patients aged 0–18 years who received treatment in CCHE during the period 2013–2017. Results showed that the median cost for a childhood cancer patient was $14,774 at 1 year post-diagnosis, and $19,799 at 3 years post-diagnosis, with ALL, AML and neuroblastoma accounting for 53.1% of the total costs. Because CCHE is a state-of-the-art childhood cancer treatment centre, not only in Egypt but in the whole region and continent that offers treatment regimens and services often unavailable in other centres, these results cannot be generalised to the rest of Egypt.

#### Cost-effectiveness estimates

Costing studies using local cost data serve as the scientific basis for accurate and reliable inputs for determining the cost-effectiveness of childhood cancer treatment [34]. Thus, the costing study by Soliman *et al* [33] served as a basis to conduct a retrospective cohort study to estimate the cost-effectiveness of childhood cancer treatment for all cancers combined and stratified by ICCC-3 groups, risk/stage at diagnosis and disease status (relapsed, refractory or progressive) [35]. This study highlighted that, for all cancers combined, the cost per disability-adjusted life year (DALY) was $1,384, which was approximately half the gross domestic product (GDP) per capita, with Hodgkin’s lymphoma and retinoblastoma being the most cost-effective cancer type to treat, and high-risk acute leukaemia and high-risk neuroblastoma being the least cost-effective. Because the treatment of r/r childhood ALL and AML was not cost-effective in CCHE, our group conducted a systematic review of the costs and cost-effectiveness of treatment for r/r childhood ALL and AML to address the gap in systematic evidence from the literature [36].

### Facilitators and barriers

Many of the barriers that exist in LMICs also exist in Egypt. The top three barriers to childhood cancer care in Egypt are delays in diagnosis leading to advanced-stage at presentation, deaths due to infections and treatment-related toxicities and non-adherence to therapy [16, 37]. Around 50% of children with cancer in Egypt experience delays in diagnosis, with a median delay of around 37–49 days from the start of symptoms until initiation of treatment [38, 39]. In addition to the facilitators in LMICs, the establishment of CCHE in 2007 has facilitated the provision of comprehensive paediatric oncology care in one place, with dedicated multi-disciplinary teams [21]. Because CCHE treats children with cancer free of charge, treatment abandonment due to financial hardships and inability to pay is reduced [16].

A qualitative study was conducted using semi-structured interviews to understand the clinicians’ perceptions of barriers and facilitators to implement cost-effective, evidence-based treatment for children with cancer in Egypt [40]. Based on the input of 14 participants, 9 paediatric oncologists, 3 surgeons and 2 radiation oncologists; the main barriers included the absence of readily available costs/cost-effectiveness data, limited resources and inability to pay for expensive novel (cost-effective) drugs and a gap between evidence and practice. However, the main facilitators included adopting standard treatment protocols based on clinical effectiveness, leadership support, availability of patients’ clinical and cost data from the local context, and existing knowledge and skills in clinical research and health economic evaluation [40].

## Discussion

This study provides a situational analysis of the status of childhood cancer care and outcomes in Egypt. To take steps towards accomplishing the GICC goal of achieving a 60% net 5-year survival rate in 2030, we need to clearly define the starting point and then set feasible milestones to achieve the goal. This narrative review has shed light on multiple gaps in the available evidence, which our research group attempted to address by publishing a series of peer-reviewed articles in recent years using data from CCHE [22, 33, 35, 36, 40]. Given that other childhood cancer centres in Egypt are probably very different from CCHE, with different outcomes, resource utilisation, barriers and facilitators, there is a need to streamline data collection on a national level, align treatment guidelines, and transfer expertise from centres of excellence (such as CCHE) to others.

The GICC introduced the Cure*ALL* framework to help countries improve their childhood cancer care. The acronym summarises four pillars (Centers of Excellence, Universal health coverage, Regimens and roadmaps for diagnosis, Evaluation and monitoring) and three cross-cutting enablers (Advocacy, Leveraged financing and Linked governance) [3]. Amongst most LMICs, Egypt stands out because it has CCHE, a centre of excellence that treats nearly half the patients while following international regimens and implementing comprehensive monitoring and evaluation programs. Thus, it is safe to consider Egypt to possess all Cure*ALL* pillars, to at least some extent. This notion can be validated by the findings of the SIOP global mapping program on the economic and population indicators in paediatric cancer care in Africa [11]. Findings showed that, of the 48 African countries participating in the exercise, Egypt was one of nine that reported availability of paediatric oncologists, chemotherapy, surgical expertise and radiotherapy. Moreover, it was one of only four that reported the availability of specialised paediatric surgery. SIOP mapping program also underscored Egypt as one of the few countries in Africa providing universal health coverage [11].

In efforts to maximise the effective role of CCHE/Egypt in extending the GICC activities of the CureALL framework to other African and Arab countries, CCHE initiated effective partnerships and collaborations with professional institutions and governmental bodies in the region. Some collaborative efforts in Africa include partnership with the Ministry of Health in Guinea for professional development and capacity building at CCHE/Egypt [41]; conducting twinning programs with major African hospitals and academic institutes to disseminate the knowledge and resources of CCHE through training healthcare professionals (oncologists, nurses and pharmacists) at CCHE/Egypt [42]; and organising the 3rd African Continental Meeting of SIOP which was held in March 2019, in Cairo/Egypt [43]. Other collaborative efforts with Arab countries in the EMR include CCHE being designated as one of the authorised training sites in the region by the *Pediatric Oncology East and Mediterranean* group to share CCHE knowledge and resource for capacity building in the EMR [44], and partnership with the Iraqi Cancer Board to train nurses at CCHE [41]. However, some challenges limit CCHE’s capacity to extend the GICC activities to other countries in the region, such as limited funds and resources, political conflicts in some countries in the EMR and the need for global commitment and direct involvement of international bodies (e.g.: WHO and SIOP) to coordinate such efforts on a larger scale.

Several key factors must be prioritised to foster the three cross-cutting enablers in Cure*ALL*. For example, advocacy needs to be backed up by scientific evidence, which will not be available unless vital data such as incidence and survival are collected uniformly from all centres on a national level in a population-based registry. Our findings showed that incidence figures are usually retrieved from two modelled sources, which rely on a set of simplifying assumptions. Unfortunately, the population-based cancer registry program, launched in 2008, focused on a small fraction of the population, omitted collecting survival data and was likely discontinued after that. Leveraged financing requires multistakeholder involvement. Although the non-profit sector plays a major role in funding childhood cancer services in Egypt, other stakeholders, including the government, should join forces to ensure sustainability and endure challenging economic crises. Moreover, **l**inked governance must be pursued to align and harmonise efforts across all centres. This will hopefully be facilitated and expedited given that Egypt recently joined the GICC as a focus country after the Ministry of Health expressed interest in prioritising childhood cancer care.

The *Lancet Oncology* Commission on Childhood Cancer estimated that 6.2 million deaths, which is more than half the projected number, could be averted between 2020 and 2050, producing a gain of 318 million life-years and $2,580 billion global lifetime productivity gains if funding were invested to scale up cost-effective interventions [1]. This would result in a net return of $3 for every $1 invested [1]. An additional aspect to consider is the family spillover effect. Evidence demonstrates that parents bereaved by cancer often experience increased rates of depression, posttraumatic stress disorder and debilitating grief that commonly persists and even intensifies after the first year, often leading to divorce [45, 46]. This leads to even further productivity losses. Thus, from a strict business perspective, treating childhood cancer has a high return on investment. However, from an equity perspective, it is inhumane to label childhood cancer as unaffordable to treat in LMICs without attempting to gather the necessary evidence and design a feasible NCCP that includes comprehensive childhood cancer care. In the past few decades, thanks to scientific breakthroughs, childhood cancer has switched from being uncurable to a curable disease in many instances, and the global community should capitalise on this privilege.

Our recommendations to multistakeholder groups leading the childhood cancer services in Egypt as they work together towards improving services are to take the following 15 actions.

1. Start a dialogue with all stakeholders to identify key opportunities and align efforts in the most efficient manner.
2. Create a population-based cancer registry that includes data regarding incidence, treatment outcomes and survival that encompasses all governorates, facilities and types of patients.
3. Design an NCCP with a section designated to childhood cancer which also addresses finances, budgeting and logistics.
4. Establish national diagnostic and treatment guidelines, encompassing several tiers of care to suit the resource availability of different facilities in the country.
5. Educate the public about the early signs and symptoms of childhood cancer, and stressing the importance of early diagnosis.
6. Ensure the existence of competent referral systems, launch initiatives to minimise treatment abandonment and track patients throughout the treatment journey.
7. Leverage the presence of centres of excellence such as CCHE, to transfer expertise to other centres throughout the country.
8. Dedicate sufficient funds to ensure the sustainability of services despite economic crises and minimise out-of-pocket expenditures including indirect costs, which might lead to financial hardship and treatment abandonment.
9. Collect and analyse costing data regularly from the local context.
10. Conduct cost-effectiveness analyses periodically, to identify and adopt highly cost-effective approaches and to reassess non-cost-effective ones.
11. Use a fair cost-effectiveness threshold to assessing interventions. A recent ISPOR abstract by an Egyptian taskforce proposed a country-specific threshold of 1–3×GDP/capita, with an additional 1.5–3 times multiplier for orphan medicines and a two times multiplier for the private sector compared to the public [47]. A more recent modelling study proposed a more stringent threshold of 34% of the GDP/capita for Egypt, calculated based on growth in life expectancy and health expenditures [48].
12. Assess barriers and facilitators of providing and receiving cancer care by collecting data from the healthcare providers and the patients and/or their caregivers, and implement modifications based on these findings.
13. Construct a database specific to evidence-based clinical practices related to childhood cancer to ensure healthcare providers stay up to date with the most novel breakthroughs.
14. Explore the potential of Egypt serving as a regional hub by becoming a referral site to neighbouring countries that might not have the capacity to foster the same level of expertise.
15. Tap into the wealth of benefits brought by participating in clinical trials, which is restricted due to legal constrictions.

## Conclusion

Egypt has the potential to become a beacon of hope and a role model to other LMICs, owing to its achievements in the childhood cancer care sphere, and its unique geographical location. As a GICC focus country, Egypt is prepared to move towards streamlining all efforts nationally and envisioning a strategy that sets clear and feasible milestones to advance childhood cancer care while being inclusive and considerate of the specific needs of different stakeholders.

## List of abbreviations

ALL: acute lymphoblastic leukaemia; AML: acute myeloid leukaemia; CCHE: Children’s Cancer Hospital Egypt; EMR: Eastern Mediterranean Region; GICC: Global Initiative for Childhood Cancers; GDP: gross domestic product; IARC: International Agency for Research on Cancer; ICCC-3: International Classification of Childhood Cancer, 3rd edition; LMICs: low-and-middle-income countries; NCCP: national cancer control plan; NCRP: national cancer registry program; r/r: relapsed/refractory; SIOP: International Society of Paediatric Oncology; TRM: treatment-related mortality; WHO: World Health Organisation.

## Conflicts of interest

RS was supported by Egypt Cancer Network (ECN). CH is funded by the National Institute for Health Research (NIHR) School for Primary Care Research [Project Number 390] and NIHR Oxford Biomedical Research Centre. CH received expenses for his media work and the WHO. NSB was supported by ALSAC. The views expressed are those of the author and not necessarily those of the funding sources. All remaining authors report that there are no competing conflicts of interest to declare.

## Funding

This work was supported by Egypt Cancer Network (ECN); NIHR School for Primary Care Research [under Project Number 390]; and ALSAC. The funding sources played no role in the study design, data collection, analysis, or decision to submit the work for publication.

## Figures and Tables

**Table 1.** MEDLINE search strategy.

Topic	Search strategy
Childhood cancer burden and outcomes in Egypt; disease burden; survival	‘burden’ OR ‘incidence’ OR ‘mortality’ OR ‘survival’ AND ‘childhood cancer’ AND ‘Egypt’
Childhood cancer treatment in Egypt; resources; costs; cost-effectiveness	‘treatment’ OR ‘management’ OR ‘resource’ OR ‘costs’ OR ‘cost-effectiveness’ AND ‘childhood cancers’ AND ‘Egypt’
Barriers and facilitators to paediatric oncology care in Egypt	‘barriers’ OR ‘facilitators’ AND ‘paediatric oncology’ AND ‘Egypt’
